# Case of Suppurative Pericarditis

**Published:** 1876-04

**Authors:** 


A Case of Suppurative Pericarditis.—This case, occuring in a patient 13 years of age, was supposed to be secondary to a pleurisy, and was probably developed by the admission into the pericardium of a trifling quantity of pleuritic effusion through a minute opening, although no evidence of such opening was found at autopsy.

Pus to the amount of thirty ounces was removed from the pericardial sac by means of the aspirator, previous to death, and thirty-six ounces were found remaining at the post-mortem examination.

With regard to aspirating the pericardium, the conclusion arrived at was, that this operation may be resorted to as palliative, but not as a curative measure.—*Ex.*

